# Conflicted Anger as a Central Dynamic in Depression in Adolescents—A Double Case Study

**DOI:** 10.3390/ijerph18126466

**Published:** 2021-06-15

**Authors:** Arne Kristian Henriksen, Randi Ulberg, Bjørn Peter Urban Tallberg, André Løvgren, Hanne-Sofie Johnsen Dahl

**Affiliations:** 1 Department of Mental Health and Addiction, Child and Adolescent Psychiatry, Østfold Hospital Trust, P.O. Box 300, 1714 Grålum, Norway; Peter.Tallberg@so-hf.no; 2 Institute of Clinical Medicine, University of Oslo, P.O. Box 1039, Blindern, 0315 Oslo, Norway; randi.ulberg@medisin.uio.no; 3 Research Unit, Division of Mental Health and Addiction, Vestfold Hospital Trust, P.O. Box 2169, 3125 Tønsberg, Norway; h.s.j.dahl@psykologi.uio.no; 4 Department of Psychiatry, Diakonhjemmet Hospital, P.O. Box 85, Vinderen, 0319 Oslo, Norway; 5 Division of Mental Health and Addiction, Oslo University Hospital, P.O. Box 4959, Nydalen, 0424 Oslo, Norway; andre.lovgren@medisin.uio.no; 6 Department of Psychology, Faculty of Social Sciences, University of Oslo, P.O. Box 1094, Blindern, 0317 Oslo, Norway

**Keywords:** teenagers, psychodynamic features, qualitative research, case studies, transference

## Abstract

The incidence of depression in teenagers has increased for many years and is one of the most common diagnosis in adolescent psychiatry. Effective and accessible psychotherapy methods need sustained attention since psychopharmaceutic treatment might be less effective in younger people than in adults. The First Experimental Study of Transference–In Teenagers (FEST-IT) is a Randomized Controlled Study (RCT) with a dismantling design. The main intention in this study was to illustrate a way to address parts of a case formulation by focusing a psychodynamic feature in two different therapies with a good outcome suffering from depression. We present two representative patients from the FEST-IT with case formulations revealing conflicted anger. The patients were different in many aspects, as were the therapeutic methods. Therapies with and without transference interpretations may help to understand what is helpful in therapy in general. It may also show how a more individualized approach can guide the therapy beyond diagnosis and to make it more effective for the specific patient. Looking into individual cases with good outcomes can help us address dynamic features in therapy and give some ideas about what works for whom. The use of nested qualitative double case studies may together add more knowledge about working aspects in successful therapies.

## 1. Introduction

About a quarter of the world’s population is 10–24 years old. The cause of years lost because of disability (YLD) in this age group is 45% due to neuropsychiatric disorders, including depression, worldwide [1]. The incidence of depression in teenagers has increased for many years and is one of the most common diagnosis in adolescent psychiatry [2,3]. The prevalence of lifetime depression is 23% among adolescents aged 12–15 [4]. Suicide, persistent and chronic mental health disorders, drug abuse and failure to achieve educationally and at work are common consequences of major depressive disorder [5]. Suicide, often as a result of depression, is the second most common cause of death at 15–24 years old (ACOG committee opinion, 2017) [6]. It is also found that adolescent depression enhances the risk of later depression and impaired psychosocial functioning in adult life [7]. Effective and accessible psychotherapy methods need sustained attention since psychopharmaceutic treatment might be less effective in younger people than in adults [8,9]. These factors call for high priority in identifying and treating depression in adolescents.

Midgley & Kennedy (2011) [10] evaluated, through a meta-analysis, the efficacy and effectiveness of psychodynamic psychotherapy for children and young people. This review suggests that there is an emerging evidence supporting the effectiveness of psychodynamic psychotherapy for children and young people. The included studies were small-scale, and they often lacked carefully selected control groups, thus making it difficult to draw any firm conclusions with confidence. Abbas and colleagues (2013) [11] completed systematic reviews of psychodynamic-based psychotherapies. This review suggests that short-term psychodynamic psychotherapy (STPP) may be effective in children and adolescents across a range of common mental disorders. According to Abbass and colleagues, there is an increasingly strong evidence base emerging for the treatment, especially for children and young people with depression [11]. It is also found that adolescent depression enhances the risk of later depression and impaired psychosocial functioning in adult life [7].

Building on these systematic reviews Midgley and colleagues (2021) [12] made an updated literature search, covering the period between January 2017 and May 2020, and a narrative synthesis of these new studies with those identified in the earlier reviews. The updated search identified 37 papers (28 distinct studies). The narrative synthesis of findings indicates that there is evidence of effectiveness for psychodynamic therapy in treating a wide range of mental health difficulties in children and adolescents. The evidence suggests this approach may be especially effective for internalizing disorders such as depression and anxiety, as well as in the treatment of emerging personality disorders and in the treatment of children who have experience of adversity. Although the number of studies is still very small compared to other treatment modalities, there is now a growing evidence-base that suggests that psychodynamic therapies can be effective for children and young people presenting with a wide range of clinical issues [12]. Hayes and colleagues (2021) [13] conducted a systematic review of shared decision-making interventions in child and youth mental health. Most studies scored low on the quality assessment criteria, as well as having a small number of studies included and a low number of behaviour change techniques utilized, and links between behaviour change techniques, intervention functions and increased participation remain tentative. Intervention developers and clinicians may wish to consider specific intervention functions and behaviour change techniques to facilitate shared decision making [13].

Less is known about how therapy works for the individual and what the helpful factors are in psychotherapy for young people. To further explore how therapy works, qualitative methods are suitable and requested [14,15,16]. Adolescents are in a crucial stage where how to process emotions and acquire a better ability for abstract thinking are changing and under development. They live more in the here and now and may have less focus on the past and the future [15]. During this period, young people might be thinking and behaving more concretely. Older adolescents can think more hypothetically, deal with ambiguity and share thoughts about the future. They may also share complex underpinnings of emotions and stressful events [17,18,19]. To establish alliance with adolescents often differs from alliance building with adults. The adolescents are at a stage in their development in which they struggle with autonomy and individuation. Their dependency upon their family signifies the importance of coping abilities and their adaptive strength. The formation of individual standpoints is a developmental task of adolescence and include plans including career, personal life and important life goals of young people. Further, adolescents are more seldom the ones to ask for treatment themselves. The age difference with their therapist might be large, which might make it difficult for them to connect with the therapists. Therapists may also be seen as just another authority figure in their lives [7]. The adolescents live in another cultural landscape than their therapists’ coming of age. We need more knowledge about how therapists address these issues, and how young patients evaluate them.

To bridge the gap between symptoms and treatment, the construction of a case formulation may be essential for a successful therapy [20]. A psychotherapy case formulation is essentially a set of hypotheses about the “causes” and precipitants, and maintaining influences of a person’s psychological, interpersonal and behavioral problems and personal strengths. The formulation may help the therapist to organize complex clinical phenomena that may guide the therapist in selection of treatment components and techniques [21,22,23]. In creating a thorough description of a patient, the therapist aims to work out a plan for the use of the most suitable interventions for the specific patient [14]. The process of developing unique case formulations for individual patients may be necessary for therapeutic change, and to develop new patterns in their life [14,24,25]. Davies and colleagues (2020) [26] conducted a study about the trajectories of depression symptom change during and following treatment in adolescents with unipolar major depression. A fast reduction in depressive symptoms in the first few weeks of treatment may not indicate a good prognosis. Halted improvement is only seen after 18 weeks of treatment. Longitudinal modelling may improve the precision of revealing differential responses to treatment. Improvement in depressive symptoms may be somewhat better in the year after treatment than previously considered [26].

A personalized case formulation may result in a more effective psychotherapy. The therapist and patient developing the case formulation together and focusing on the predefined themes during therapy may also strengthen motivation and hope in achieving changes [27,28,29]. To understand the patient’s needs and treatment goals and to develop an idiosyncratic, patient-centered formulation may be necessary to guide a successful treatment. Agreement between a client and his or her therapist regarding how to understand the client’s illness (pathogenesis), as well as what might be helpful in therapy (cure), may motivate and empower the client to work on his or her issues [24,30,31,32].

Busch and colleagues (2004) [33] developed a method to establish structured case formulations as a mean to improve psychodynamic therapy for patients with depression. The described factors in the model are based upon a depiction of symptoms and problems, precipitating stressors or events, predisposing life events or stressors. This model for development of case formulations has been adopted in the British study, Improving Mood with Psychoanalytic and Cognitive Therapies [15,34], and in the First Experimental Study of Transference–In Teenagers (FEST-IT) [35]. Focused short-term treatments based on personalized case formulations may result in a good outcome, even in cases with patients with severe psychopathology. The results may also last over time [36].

The Central Dynamics of Depression (CDD) described by Busch and colleagues (2004) [33] are defined as narcissistic vulnerability, conflicted anger, severe superego, idealized and devalued expectations of self/others and characteristic means of defending against painful affects.

*Narcissistic vulnerability* describes an insecure sense of a separate self and a heightened sensitivity to perceived or actual losses and rejections leading to a lowering of self-esteem, which in turn triggers depressive affects, existential anxiety, and rage in response to narcissistic injury.

*Conflicted anger* describes anger, blame, and envy directed toward others that leads to disruptions in interpersonal relationships, confusion over responsibility and to self-directed anger and subsequent depressive affects.

*Severe superego* describes experience of guilt and shame that may impact feelings and wishes seen as bad and/ or wrong, with doubt about whether the young person’s love outweighs aggression, leads to negative self-perceptions and self-criticism and in some cases confusion between reality and fantasy.

*Idealized and devalued expectations of self/others* describe high self-expectations and/or idealization of others, often switching to sudden de-idealization and devaluation that may lead to disappointment, anger at self and others, and subsequent lowering of self-esteem.

*Characteristic means of defending against painful affects* describe use of defenses typical to depression such as denial, projection, passive aggression, and reaction formations. This may lead to increased depression because either the world is seen as hostile or the self is attacked.

Splitting, as a characteristic defense against aggression, blocks assertive/aggressive efforts from integration in the service of personality development [33].

Narcissistic vulnerability has been described as a Central Dynamics of Depression (CDD) among clients that may trigger suffering in patients with depression. Struggle might originate from disappointments in childhood relationships and to other developmental experiences leading to a fragile self-esteem. Narcissistic injury may also predispose individuals toward the experience of shame and anger, which later on may trigger a depressive state. Conflicted anger might be another trigger. Anger is sometimes triggered by narcissistic injury, but it may also be the result of loss, frustration or a sense of helplessness.

Trowell and colleagues (2003) [37] found more suppressed anger in individuals recovered from depression than in healthy controls, along with an increased fear that expressing anger could damage relationships. Luutonen (2007) [38] have indicated either an increase in outwardly directed anger or a greater degree of suppressed anger in patients with depression. Goldman and Haaga (1995) [39] found increased anger, suppressed anger and fear of expressing anger in self-reports of outpatients with depression compared with controls without depression. Wolfersdorf and Kiefer (1998) [40] showed that, compared with healthy controls, in-patients with depression had increased levels of inhibited aggression and covert hostility, but did not express aggression. Conflicted anger seems to be a prominent feature of depression.

The use of mediators and moderators in youth psychotherapy and the way therapy is brought through are poorly understood [10]. The use of a case formulation as a supplement to formal diagnosis may guide and focus treatment. If conflicted anger is agreed upon among the patient and therapist as the trigger of depression, it may also give some ideas about how anger and depression may interact and how it can be explored during therapy. The agreement of a personalized case formulation may be decisive to how the client benefit from treatment [24,30,41].

The clients’ recovery in treatment may be shown by the fulfillment of expected goals in treatment but may also be shown by changes in core relational themes (CCRT) and in the co-created stories toward the end of treatment. The search for a change may be an open-minded and challenging enterprise. How far did we come in therapy, and how do we prepare the client from upcoming struggle and events during the final sequences in therapy and post treatment? How the therapy corresponds with their symptom reliefs, the clients’ values and their sense of coherence may be decisive. The use of case formulation from the beginning of treatment may together with the chosen outcome measures consolidate therapeutic work as well as preparation for the ending of therapy [7,42,43].

Core Conflictual Relationship Theme (CCRT) is a method describing relationship patterns in case formulations [44]. Slonim and colleagues (2011) [45] examined changes in the rigidity of interpersonal patterns and symptoms in adolescents (ages 15–18) in one-year psychodynamic psychotherapy. Adolescents in the treatment group became less rigid in their interpersonal patterns and improved significantly in their symptoms. In a follow up study [46] the adolescents’ positive representations of their therapists increased. Their findings were also related to changes in their representations of their relationship with their parents and to treatment outcomes. There was an association between the development of the therapeutic relationship and improvement in the perception of the relationship with parents over the course of therapy. Therapists who look for psychologically derived and culturally embedded interpretations for intellectual and emotional distress may provide interpretations that are adaptive and accepted by the patients.

## 2. Aims and Objectives

The systematic reviews and the narrative synthesis of findings indicates that there is evidence of effectiveness for psychodynamic therapy in treating a wide range of mental health difficulties in children and adolescents. The introduction address central dynamics that may contribute to the understanding of depression in adolescents. It also highlights the importance of therapists’ active using the case formulation in the treatment of depression. In the present study, we focus on how a specific psychodynamic feature described in the case formulation, namely conflicted anger, guided the therapists’ work in two good outcome therapies. How can it improve outcome to address central dynamics of depression in psychodynamic therapies with and without transference work in the two therapy modes in FEST-IT? How the client and the therapist work together following a case formulation based on clinical information and psychodynamic theory, may add to the growing knowledge base on individualized treatment. The use of conflicted anger as a dynamic feature in the treatment of depression in psychodynamic therapies may provide some new knowledge from case studies.

## 3. Materials and Methods

FEST-IT is a Randomized Controlled Study (RCT) with a dismantling design. The patients were randomized to one of two psychodynamic treatment modes which differed slightly in the way the therapist encouraged the patient to discuss interpersonal relations. In the transference therapy, the therapist encouraged the patient to explore the relationship with the therapist (transference interventions). In the non-transference therapy, this was not specifically encouraged by the therapist. In both therapies, the patient’s relationships with other people were explored. The most frequent mechanisms, based on therapist reports for depression in this study, were narcissistic vulnerability 31%, severe superego 31%, and conflicted anger 22%.

In our study, two patients from the FEST-IT study with good outcomes in therapy were selected to illustrate how conflicted anger triggers depression and how it can be worked with in therapy. Both patients were evaluated with conflicted anger as their CDD. The two patients, one male and one female, were randomized to each one of the treatment arms [47]. FEST-IT is a randomized, controlled study on psychodynamic psychotherapy for adolescents with depression. The Central Norway Regional Ethics Health Committee approved the study protocol (2011/1424 FEST-IT). FEST-IT is registered in ClinicalTrials.gov: NCT01531101. All participants signed a written consent.

In the present study, a qualitative method is used to examine contemporary real-life situations and apply the findings of the case to the predefined problem under study. A case study involves a detailed contextual analysis of a limited number of events or conditions and their relationships. By using an intensive approach about a person, a group or a unit, the study aims to generalize data over several units. The first component of the process is usually the selection of either a single or a multiple case design [48,49]. Benefits of the use of multiple cases include the opportunity to analyse the data both within each situation and across situations. By focusing on similarities and differences between cases, the analysis may discuss issues from theory as well as potential research questions.

### 3.1. Patients

69 adolescents aged 16–18 years diagnosed with unipolar major depressive disorder (MDD) according to Diagnostic and Statistical Manual of Mental Disorders, Fourth Edition (DSM-IV; American Psychiatric Association, 2000) [50] were included in FEST-IT. Axis I and II diagnosis were based on the Mini International Neuropsychiatric Interview [51], and Structured Interview for DSM-IV Personality [52] respectively. For pre-treatment characteristics, please see Table 1.

### 3.2. Treatment

The treatment was short-term psychodynamic psychotherapy (STPP) over 28 weeks, with one session a week lasting 45 minutes, employing the STPP treatment manual from the IMPACT study [34]. The manual focuses on techniques aimed at helping young people overcome developmental and relational problems as well as emphasizing the role of the interpretation of unconscious conflicts, insight, and the concepts of internal working models. The manual combines aspects of STPP that focus principally on techniques aimed at helping young people overcome developmental problems, as well as emphasizing the role of the interpretation of un-conscious conflicts, attachment theory and the concepts of internal working models. In FEST-IT, patients were randomized to one of two treatment modalities, either with transference interventions or no transference interventions. In both groups, the interventions of both treatment modalities were directed at exploring the adolescent’s interpersonal relationships as well as the thoughts and feelings that the adolescents may avoid, and paying attention to repetitive patterns of thoughts, feelings, and actions. In the treatment modality applying a moderate level of transference work (i.e., one to three per session), the therapists prescribed additional interventions that in different ways addressed the dynamic of the patient-therapist relationship in the here and now. All therapy sessions were audio-recorded.

### 3.3. Assessment

Two interviews; the Psychodynamic Functioning Scale (PFS) [53,54] and the Global Assessment of Functioning (GAF) [55], as well as two self-reports; the Global Severity Index (GSI) from Symptom Checklist-90 [56], and the Beck Depression Inventory (BDI) [57] were completed in all cases. The patients were interviewed by an evaluator before, after, and 1 year after treatment. In addition, SCL-90 and BDI was scored after Session 3, 12 and 20. During the initial PFS interview, the patient in detail described his or her relationships to parenting figures, friends and other important persons in their lives. They also described how they handled difficult situations in their life and how they managed in stressful situations. The interviewer was seen as a co-producer of the data who, together with the participant, affected the interview process. This interactive approach meant that the data were not only seen as objective information coming from inside the patient but were also produced in a unique encounter between the persons involved, who act and react upon each other [58]. Together with the anamnestic background information, this interview informed the development of the case formulation [33]. Through this method, the patient was actively involved in course of the treatment from the start. The therapist was in corroboration with the patient, guided in how to proceed in therapy.

### 3.4. Therapists

Therapists with at least 2 years of formal education in psychodynamic psychotherapy treated the patients. To maintain the quality of the therapies and adherence to the manual, peer supervision groups were offered throughout the study period. This also was to ensure that the therapy mode in each therapy group was delivered. The therapists treated patients in both treatment groups.

### 3.5. A Nested Qualitative Double Case Study

Within the present study, the analysis was carried out as an additional in-depth analysis of qualitative data from FEST-IT, with the aim of achieving more intensive focus on a particular finding or aspect than was undertaken as part of the quantitative primary analyses [59,60,61]. We present two patients from the FEST-IT with case formulations revealing conflicted anger. Data are anonymized, and it is not possible to reconnect the clinical material to patient identity. As it was the aim of the study to learn from the cases, the chosen patients had good outcomes. One male patient treated in the non-transference group and one female patient from the transference group were selected. Thus, the patients represent different sexes and received treatment from each one of the treatment arms. Conflicted anger was the presented central dynamic of depression in the case formulation and was thought to be the underlying mechanism and trigger of the depression in these two young persons.

## 4. Data Analysis

We used a consensual qualitative approach to extract knowledge from the clinical interviews [59], and Interpretive Phenomenological Analysis (IPA) was used to analyze the therapy sessions [61]. IPA focuses on how individuals make sense of events or experiences that are associated with the topic under study and is a commonly used approach to obtain meaning from small-scale studies. The raters adopted a broad approach to capture the diversity of the adolescent’s experiences, and the phenomena present themselves as they occur in the dialogue. The amount of thick description of the phenomena under study may illustrate how the central themes are dealt with. It also grants a deeper understanding to the aims and scope of this study [22,23]. By comparing individual cases, we aimed to identify the commonalities and differences within and between therapies.

We analyzed selected data from the themes chosen by therapist and evaluator as the dominating CDD, and the presented vignettes are the extracts from the recorded therapy sessions. The parts displayed in the vignettes were decided by listening to the therapy sessions three times, with focus on parts of the dialogue between therapist and patient where either one of them is addressing the CDD. Sometimes this was done explicitly, e.g., “You never get angry?” and sometimes it is more subtle, e.g., “So when these (angry) feelings come, you kind of evaluate them?” The picked parts in the study were collected from the beginning, the middle and the final sessions. Other essential parts of the therapy sessions are excluded in this presentation, as the focus was on the described CDD. Our aim was to identify categories, core ideas and themes that were common within and between the study samples [22,23,25,61].

During the initial stages of the analysis, the therapy sessions were listened to several times while taking notes. The notes included the interviewers’ own reflections on the repetitions. Together they settled major themes from the analysis as well as their connections to theory. In the subsequent phases, the aim was to obtain a sense of the information as a whole. The clusters of concepts from each account provided the background material for the researchers to write separate text summaries of the responses of participants in both samples. The identification of core issues and CDDs in the two samples reflected commonalities and differences across the narratives. The shift to an examination of the sessions as a whole as well as the themes and categories from each account was an important step in capturing the salient features of respondents’ experiences in dyads. This procedure involved a double hermeneutic, as the researcher attempted to make sense of the participants’ attempts to make sense of their own experiences [62,63].

Looking into individual cases with good outcome may help address dynamic features in therapy and enable the comparison of categories and themes at different levels of understanding [59,61,62]. During the comparative process, the team subsumed the two meaning units described earlier into a subcategory at a higher level of abstraction. Self-scrutiny and competing interpretations from members of the research group, enabled us to identify different aspects of our personal motivations, the influence of our clinical experience, and our reliance on theoretical issues. It also enabled the comparison of categories and themes at different levels of understanding [59,61,62]. During the process, we refined the emerging subcategories, making them more comprehensive and precise [23,59,63]. During the collective process of analysis and discussions within the research group, we aimed at staying open, ensuring the transparency and trustworthiness of the results, and reducing biases [59,64].

## 5. Results

Conflicted anger was a frequent Central Dynamic for Depression (CDD) in the case formulations in the FEST-IT study (22%). For the present study, two anonymised patients with good outcome and for whom *conflicted anger* was the described mechanism for the depressive symptoms were chosen. *Conflicted anger* describes anger, blame, and envy directed toward others that lead to disruptions in interpersonal relationships, confusion over responsibility and to self-directed anger and subsequent depressive affects [33].

The patients were one female who received psychodynamic psychotherapy *with* and one male receiving therapy *without* transference work. The case reports intend to show illustrative vignettes from therapy sessions where conflicted anger is in focus. The agreement between about what should be the focus from the beginning of therapy may signify the therapists’ and patients’ adherence to conflicted anger as a central dynamic may be crucial.

### 5.1. Anna

Symptoms and problems:

Anna, a 17 soon-to-be 18-year-old female was referred to psychotherapy due to depressive symptoms during the prior two months. She described that she found it difficult to talk about herself, and that she was not used to doing so. She described symptoms compatible with depression; she felt sad, had an increased need for sleep, decreased interest in herself and others, and she felt numb.

Pre-treatment evaluation:

She lived with her father. During the pre-treatment evaluation, she said that she recently had been to her regular training at a training studio, but she did not enjoy it. The very same day as the interview, she had been invited to a friend for coffee, but she had rejected the invitation since she intended to go to her training. Afterwards, she felt lousy and decided to pause her training. One week later, she had an exam in mathematics and found it overwhelming. She felt trapped and wept a lot. She rated herself as depressed (BDI score was 24). Her global and psychodynamic functioning was reduced (GAF at baseline was 63 and PFS mean score was 65.8.

Diagnosis:

She was diagnosed with Major Depressive Disorder

Mechanism and Trigger of Depression:

Her depressive symptoms were precipitated by a conflict concerning plight and pleasure. She seemed to strive with self-assertion and to some degree of repressed aggression. The mechanism considered was *conflicted anger.*

Treatment:

She was randomized to therapy with transference interventions. No antidepressant medication was prescribed. She attended 28 sessions. In the first session she started talking about how she felt tired when she was at school:

Anna: Everybody is asking about Sam, my ex-boyfriend. They want to know if we are still a couple. It makes me feel under pressure. I get so affected by what others think or say.

Later, in the same session, she talked about an argument she had with her mother. When the therapist asked about what happened, it was difficult for her to describe the sequence of events:

Anna: I don’t remember, it just felt bad, like yesterday, everybody is asking a lot of questions.

After a few sessions, Anna reported that the symptoms of depression decreased. After session 11, Anna again talked about Sam. They were now again together. She felt that she had fallen back in the depression again. She knew that it had to do with Sam leaving her for three days:

Anna: It made me think of the next year at school, many of my best friends leave school this year and the next year they will be gone…

Therapist: It is natural that you feel sad when you are missing a friend, you must allow yourself to mourn.

The therapist was active in helping the patient to allow all her feelings, not to deny painful emotions, but to let them be there. Anna felt comforted by the therapist’s support.

After session 12, BDI-score was 22, but parallel with the patient feeling better again, at session 20, the BDI-score was 6.

In session 21, the therapist encouraged the patient to discuss the fact that the therapy was soon to end. The patient told about her plan to go to San Diego. A friend of her lived there for one year as an au pair. Anna had been invited to stay during the summer holidays. When she told a friend at school about this, the friend reminded her that they had planned to go to Turkey together, and that she herself had agreed on that. Anna was confused when she brought this up in therapy:

Anna: I can’t remember that we decided to go to Turkey. It might be we spoke about it once but nothing was decided.

She felt confused and tired.

Anna: I became sad because of what she said. I had a strong belief it would be a really nice day, because it started so very, very good. It was so irritating that the travel to Turkey came up… something I then had to consider.

Supported by the conflicted anger mechanism described in Anna’s Case Formulation, the therapist focused on anger:

Therapist: Mm. I was also thinking about that, with regard to what we have been talking about. That of that when the feeling of being sad comes, maybe there is something beyond it, like you being irritated?

Anna: Mm, I don’t know exactly what it is or what kind of feeling I have. I don’t know where I stand with this. Like, is it something I should think much about, or isn’t it a big deal anyway?

The therapist continued to try reducing the patient’s avoidance to exploring her feelings concerning anger.

Therapist: I suppose it is a situation where you actually don’t know what you feel. Is that correct?

Later on, in the same session, the therapist reminded Anna of her tendency to have difficulties in situations where she needed to make a choice and she confirmed that it is tough for her to do so. She needed to prepare for the worst. The therapist supported their agreement on anger as an important theme for her as it gave her negative expectations.

In the last session, Anna talked about a situation where she had baked buns at home. Her father told her to keep the kitchen clean. Anna got irritated because she had never left the kitchen in a mess. She felt it was an unnecessary comment only meant as an attack on her. The first set of buns was a failure, but Anna felt determined to make good and tasty buns. She went to the shop and bought more flour, yeast and butter and after some trouble, she had made excellent new buns. Again, Anna felt happy. When her father came home, he was happy over the fresh buns in their house.

Anna: Then I got so irritated! I couldn’t understand how he, the very same morning, had managed to make something so simple and positive as making buns to something negative. I was really irritated. Therefore, it destroyed the experience in the start. This was a moment of irritation. How was it that he could come and tell me I’m doing wrong? This, and that he managed to make the bun baking to something negative was very irritating but then, it blew over in the same moment.

Therapist: It sounds like it was some easier to get a hold on your reactions.

Anna: Yes.

Therapist: And that you got quite irritated.

Anna: *Mm.*

Therapist: Then it sounds like it was possible to use that energy [from the irritation] to manage the bun baking, and feeling proud of it.

Anna: I was not sure of what I needed to feel comfortable again.

Therapist: You can get a hold of it; and you can use it [the irritation].

Follow-up:

At post-treatment the BDI score was 3. At one-year follow-up she scored 0. GAF at post–treatment was 80 and at one year follow-up 85. PFS at post-treatment and 1-year follow-up were 78.1, and 82.2 respectively, indicating a successive increased psychological functioning.

### 5.2. Sven

Symptoms and problems:

Sven, a 17-year-old boy, was referred to treatment and described as a social, nice young man achieving very well at school. He started treatment in September. The symptoms had gradually emerged since Christmas. He told that he felt increasingly sad, found life meaningless, had nothing to look forward to, and had lost interest in life. He had no energy. He also described sleeping difficulties and loss of appetite. The symptoms had worsened during the summer but for the prior weeks, he had actually felt somewhat better.

Pre-treatment evaluation:

Sven told that he had moved several times and changed school three times. Now he lived in a stable foster home. His parents were reported to social authorities by neighbors when he grew up. Sven was placed in an institution when he was 13 years old, due to his miserable family situation. He stayed in an institution for one year, and lost contact with his family, relatives and friends.

During the pre-treatment evaluation, he described feelings of loneliness and helplessness during his stay at the institution. He had endured bullying and watched his younger brother being beaten up by other teens. Since he moved to the foster care, he has met his parents a few times a year. None of his three siblings live together.

PFS at baseline was 57.6, GAF 53, and BDI 44.9.

Diagnosis:

He was diagnosed with Major Depressive Disorder, and recurrent depressive episodes. He was also diagnosed with Depressive Personality Disorder.

Mechanism and Trigger of Depression:

Sven had been exposed to difficulties early in life as well as hasty shifts in care.

The depressive symptoms were considered to be an expression of defense against painful and unpleasant feelings. It could be that repression, possibly projection and passive aggression, had increased his depressive symptoms. It seemed as if life events had never been worked through and maybe upheld by a biological family being fragmented for reasons Sven didn’t know of. Sven presented aggressiveness directed inwards resulting in depressive affects. This could be considered as aggressiveness directed towards others being turned inwards. The mechanism considered was conflicted anger.

Outcome

The therapy

The therapy starts with the patient feeling tired, disappointed over what he cannot achieve at school. He feels depressed and sad. He describes a feeling of emptiness. He is intent and determined to do well at school in spite of his depression or any other illness and is now really disappointed when he does not manage well enough at school.

In the fourth session, the therapist addresses his conflicted anger:

Therapist: You just get tired, never angry?

The focus thereafter aims on why the patient is afraid of showing or feeling aggression. The patient describes an unpredictable father who sometimes went mad about things, sometimes out of the blue.

In the next session, which was just after Christmas, Sven remembered Christmases in his early childhood. He talked about his parents being suspicious and how this may have made him always alert and suspicious or afraid.

After session 12, he scored BDI at 9.

In the 20th session, the patient is reminded on the time left in therapy:

Therapist: After today we have eight more sessions before the therapy is finished.

Sometime later in the session:

The therapist strives to overcome with Sven’s reluctance to talk. He focuses on Sven’s feelings and specifically on his anger.

Therapist: Yes; values, meanings, opinions (….) and then, as you know, we are also motivated by feelings.

Sven: *Mm.*

Therapist: To think logically and rationally might be helpful but often it is the feelings that drives us to choose as we do.

Sven: *Mm.*

Therapist: How much do you think about such things? Is this a part of you, something you try to investigate?

Sven: Yes, I experience some feelings and I think about it. I ask myself how to fit feelings with rationality and rationality fit with feelings.

Therapist: Yes, please tell me what you think about that.

Sven: I think it is about to manage making clear and logic choices. Eh, if it don’t crash with the feelings at the same time (…) I also try to make the feelings fit with what is logic, so you don’t get messed up with meaningless things.

Therapist: *Yeah?*

Sven: If you do that, you just use a lot of energy without meaning.

(……….)

Therapist: Yes, but then you can consider your feelings and think: ‘Now I’m afraid’ or ‘Now I’m angry’, or ‘Why am I …?’ Is this the way it is, or is it more like you feel: ‘This doesn’t matter; I have to stop that feeling’?

Sven: Well, it is more like if I am really angry with someone I first think about why. Then I consider whether it’s helpful. If it is about minor things, I just let it go. Then it just passes, and I don’t think much more about it.

Therapist: So when feelings come, you in a way evaluate them. Is this the way you think?

Sven: Yeah.

(….)

Sven: I cannot use feelings of emptiness, but anger can be constructive and motivate me to finish something. Sometimes anger also help me to develop thoughts and understanding.

Therapist: *Mm*.

Sven: In a way, it gives energy.

After session 20, Sven rated BDI at 6. At post-treatment BDI was 3. He did not meet for follow-up evaluation at post-treatment or 1-year follow-up

## 6. Discussion

The empirical literature on the association between changes in internal representations of relationships and symptom reduction is inconclusive. Although some studies have found this association to be significant [44,45,46,65], others have not [66]. McCarthy and colleagues (2008) [67] suggested that these contradictory findings might be attributed to different measurement techniques. Still, we need more studies to examine the theoretical psychodynamic assumption that changes in internal representations are associated with changes in symptomatology and outcome. In the presented study, we have analyzed two successful therapies from the FEST-IT study. The focus on central dynamics in depression as part of a psychotherapeutic case formulation may bring forth the maintenance or precipitation of further suffering. It may also address how it may increase the chances of good outcomes in psychotherapy.

The therapy with Anna was, according to GAF scores and BDI scores, successful. The fact that the BDI score stayed low even at one-year follow-up may indicate that the patient now is better off concerning earlier vulnerability to depression–or at least shows continued progress. Initially Anna struggled a lot to feel accepted by herself and others. In the first sessions, she said she paid attention to everybody who was asking her questions all the time. She also asked the therapist not to pressure her with all those questions. Anna was getting better after a few sessions. In addition, as the final session gives a hint about, the patient was during the follow up more confident in herself and possibly more of an agent in her own life. Studies from patient feedback research shows that early positive trajectory features as a rule predict good outcome [68,69,70].

In the therapy with Anna, the therapist early on in therapy addressed the chosen mechanism of conflicted anger as a dynamic feature. In the presented parts of the dialogue between patient and therapist, there are examples of preparatory transference work [71]. The therapist addresses herself in relation to the patient: “*Mm, I also thought of that, relating to what we have been talking of, when you start feeling sad. Is there sometimes maybe something more to it… that you are irritated?*” In this specific therapy, the therapist was active in using transference work, and it seems like it helped the patient forward. TI might also be considered as extra helpful for this patient, since she has trouble with relating to her father (her therapist was a man). In therapy, she directly related to the therapist in another manner, and she became gradually more aware of her own aggressive feelings. As shown in the extracts from the last session, these feelings now give her energy instead of confusion or depressive feelings and thoughts. The therapist’s active comments on their relation in therapy may be decisive for her progress. According to findings from Norcross and colleagues (2006) [72], the introduction of differences (additional, corrective or new) can serve as a source of creativity, strength and progress if constructively harnessed.

The therapy with Sven was also successful. In Sven’s therapy, the therapist presented psychodynamic features, focusing on aggressiveness early on in therapy. “*You just get tired, never angry?*” The patient struggled with his emotions, and he had a lot of trouble with listening to his feelings or considering them as much as he did with reason. In the therapy, the therapist minimalized the use of TI. The therapist just commented on what the patient brought into therapy, and on what happened to Sven himself. At the beginning, the patient was just focusing on what he achieved at school—an arena on which he earlier was successful.

During treatment, he experienced an increased sense of mastering and control in a life full of unpredictable events. After a couple of months, he was more able to attend school and perform there. Gradually, he subdued his troubles about school, and he focused a bit more on other parts of life. He started to work through painful memories, and during the later sessions, he was able to let out and trust his feelings more than in the start of therapy. In the extract from session 20, the therapist, with some effort and the use of formulations, helped the patient to explore more about how he deals with feelings and how he could use them, and not just rely on reason.

A qualitative interview study nested within the FEST-IT study explored how eight adolescents experienced improvement from depression [73]. Some of these patients emphasized working with their feelings as more important than focusing on their thinking. Others experienced the opposite. Achieving insight about their own feelings became easier when they became more aware of their thinking. Four main themes that may be helpful for a successful outcome were revealed, presented as exploring oneself, therapist relation and characteristics, their focusing on everyday life, and time factors [73]. The treatment of patients in a conflicted anger perspective in our study, may de-emphasize more expanded experiences from the patient about experiences of recovery. Anna and Sven’s descriptions of their relationships to parenting figures, friends and other important persons in their lives and how they managed in stressful situations may be decisive. Their work with predisposing life events and psychodynamic feature in their daily life may be important.

The patients’ participation in treatment from the beginning and during therapy provided opportunities to address theirs conflicted anger as a dynamic feature. Both clients appreciated tasks where they could explore both their strengths and difficulties in the social interaction with others. According to findings from Busch and colleagues (2004) [33], the ending of treatment may also be experienced as a narcissistic injury. Angry fantasies towards the therapist may intensify at this point [33]. Individuals must also confront fantasies of maintaining a special relationship with the therapist. They may also experience a resurgence of depressive symptoms as they contend with this loss. Therapists’ clinical experiences suggest that at least some patients may return to previous experiences when termination is anticipated [74]. At the end of therapy, both Anna and Sven gave examples of changes in their self-statements, and they both sounded more confident in themselves and even proud (Anna). They also shared thoughts about the future. In all, this can be understood as signs of improvement.

Both patients established healthier relationships with family and friends in therapy and post treatment (measured with PFS), and they shared a positive outlook about the future. They had achieved important goals during therapy, and they could more easily take new actions or adapt to situations that were difficult to change. They also shared thoughts about how to deal with upcoming struggles. Equipped with skills for open-minded communication and interpersonal interactions, clients may leave therapy on a trajectory of further growth. How the patients interpret themselves during therapy, their interactions in close environments and their expectations about the future may be decisive for further progress, supporting findings from Valkonen and colleagues (2011) [75]. Such experiences may also facilitate new relational experiences that can be transformative for patients [76].

As other patients in the FEST-IT study put into words how therapy contributed to improvement, they also viewed themselves in relation to expectations from family and therapists–as well as social surroundings in relation to their own needs and desires. When they gave meaning to what improvement meant, they also placed themselves in a discourse of responsible and conscientious individuals. By exploring their thoughts, emotions and actions, they could easier handle their struggle in alternative ways. They improved by talking about emotions and thoughts in therapy and beyond–and by this getting to know themselves better, supporting finding from Løvgren and colleagues (2019) [73]. They also wanted to live an ordinary life without conflicting relations or troublesome symptoms and with better relations to important others and themselves. Our study shows the participants’ need for support and help in finding their individuality and uniqueness as steps towards broader goals, supporting findings from Binder and colleagues (2010) [77].

The offered treatment in FEST-IT included weekly therapy through 28 weeks and follow-up evaluation one year after treatment termination. How the therapist best delivers suggestions to elaborate upon in therapy and when to end treatment is still underscored [27,29,78,79]. Transference work was used in Anna’s therapy, but when to elaborate talking about treatment termination may be critical. According to findings from Davis (2008) [80], therapists may find it controversial to terminate therapy if the client wants to continue therapy, or they may experience some uncertainty about the client’s further need for therapy. According to findings from Olivera and colleagues (2017) [18], it may be decisive whom that initiate the final sequences ending of therapy as well as the treatment termination. Both therapist-initiated terminations and agreed-upon terminations were associated with more categories of positive termination motives, better therapeutic bonds and higher overall satisfaction with treatment. When to end therapy may be double-edged. A fast reduction in depressive symptoms in the first few weeks of treatment may not indicate a good prognosis. Longitudinal modelling may improve the precision of revealing differential responses to treatment. Improvement in depressive symptoms may be somewhat better in the year after treatment than previously considered [26].

In a comparable study, Råbu and Haavind (2012) [81] examined the ambiguity of ending treatment. This case study draws attention to how ambiguities may be settled in a process where ending is initiated by the therapist and resisted by the client. The client and the therapist had developed a ‘good enough’ alliance (WAI), [82], and reached a ‘good enough’ outcome (OQ–45), [83], but still the client felt she was far from finished. According to findings from Anna and Sven’s therapies, a close inspection of interactional data during the final sequences of therapy elicited information about substantial content and structural aspects from the process of ending therapy. Structural elements might include preparations for a break, or reducing or intensifying the frequency of sessions, and may give some ideas about how the client managed life without therapy. Anna and Sven had come to a point where they could affirm that treatment was ending. Significantly, the final proof of improvement may be interpreted through the clients’ autonomy. The quality of the client’s participation in therapy and changes during therapy may be decisive for a successful outcome.

It is necessary to be cautious to draw any conclusions from only two cases. In observing the process in the two therapies, it looks like there is a more rapid progress in Anna’s therapy, but the patients have different backgrounds, and no conclusions can be drawn. Would Anna’s therapy been different without TI, or is TI the intervention that made a difference? From the audio recordings and the transcripts, we cannot know the answer. However, Anna was focusing a lot on relationships in her therapy, and probably she may benefit from a therapist who focuses on the therapist-patient relation. Sven had bigger difficulties in relating to his own feelings, and usually he presented his strivings in a more concrete way. Could he had been more helped in another type of therapy… as with TI? This cannot be confirmed, and for Sven it might as well have made therapy more difficult or less prosperous. Findings from studies on evaluation in therapy reveal that it is difficult for clients to give feedback if evaluations are not requested by the therapists [69,70,84].

There are many differences between the two presented cases. However, the two young people also shared common experiences. In general, the adolescents in the FEST-IT study value problem solving, and they appreciated help with concrete challenges in their life. They also improved by exploring themselves within the frames of a time-limited treatment period. Improvement seemed to be experienced through better relations to oneself and to others–and by finding their own place in the family or at school [73]. Therapeutic work was tailored to the needs of adolescents and incorporated the challenges they faced in their everyday life. The quality of the clients’ participation throughout the phases of therapy may help clients take ownership of their gains, equalize the therapeutic relationship and prepare clients for their transition away from psychotherapy, supporting findings from previous studies [27,85].

It is possible the Anna case is more explicit. It clearly shows examples from her everyday life, and she showed a trustful relation to the therapist, which helped her in exploring her own feelings, thinking and actions. Sven was maybe more restricted in exploring himself openly in therapy, but there was progress during therapy, and gradually he developed a helpful relation to the therapist. How the client participates during sequences of treatment may be decisive to how he or she engages in therapy, and how they evaluate changes during treatment. How to understand the client’s illness, as well as what might be helpful in therapy, may motivate and empower the client to work on his or her issues. It may also strengthen hope in achieving change, supporting previous findings [28,29]. Agreement between a client and his or her therapist about the case formulation and the chosen dynamic feature may be decisive [27,30].

How the patients work with the chosen central dynamic features in therapy may tailor treatment to the individual patient. During sequences of therapy, Anna and Sven had the opportunity, together with the therapist, to review the ongoing work in treatment, to summarize positive gains from therapy and identify plans for the future. Their experiences during therapy had made them stronger, and they both had established healthier relationships with family and friends. Rethinking how participants in dyads label and conceptualize helpful aspects during the final phases of treatment may help clients to engage in dynamic work as well as healthy and helpful cognitions following treatment, supporting previous findings [17,76,85,86].

When to initiate the process of ending therapy may be a critical issue in therapist-defined and agreed-upon terminations of successful therapies and require focused attention [81,87]. According to findings from Olivera and colleagues (2017) [18], the clients who did not prepare for the termination process, reported more negative emotions toward treatment. In the treatment of Anna and Sven, the therapists prepared for the treatment termination–with the aim of strengthening and reinforcing the client’s gains even if they were not fully realized. A study by Jofen-Miller and colleague (2017) [88] sought to offer greater insight into post-termination contact and the role of training and experience in shaping attitudes and behaviors. In terms of attitudes, the therapist respondents were more likely to anticipate positive rather than negative consequences of post-termination contact for both patients and themselves.

The process of ending psychotherapy may solidify improvements made over the course of psychotherapy and reorient clients to life outside of formal psychotherapy [89,90,91]. It may also facilitate new relational experiences that can be transformative for clients [42,76,80]. Picking out and putting together building pieces in a developmental story may strengthen and challenge dynamic feature in personal development [81]. Valkonen and colleagues (2011) [75] point out that the most important changes in the clients’ experiences have to do with how they interpret themselves and how they use new strategies and procedural knowledge to combat struggles in their life. This pattern may also include relational knowledge and what they may do together with others [92,93].

Even in successful therapies, there is no reason to assume that each terminated therapy process continues to move in a positive direction [81,94,95]. Stories about recovery also address periods of vulnerability and setbacks [96]. Obstacles to success following treatment remain poorly understood, and more knowledge on them is needed [97,98]. Quintana and colleague (1992) [91] compared termination in successful cases with that in unsuccessful cases. Their study demonstrated that psychotherapists in unsuccessful cases engaged in less frequent discussion of the termination phase, less review of the course of treatment, and less discussion of the client’s affective reaction to termination.

Working with central dynamics and their interactions with others may be decisive for further growth. If the patients are satisfied with their participation in therapy and their progress, they generally displayed positive reactions to the ending of treatment [31,99]. The time had come for the client to venture forward in possession of useful coping skills, self-awareness and adaptive strategies [89,100]. The question is whether clients have developed intrapsychic and relational abilities robust enough to stand the test of future difficulties and challenges in life, such as the transition into early adulthood [32,96,101]. How the client and the therapist work together following a case formulation based on clinical information and psychodynamic theory may add to the growing knowledge base on individualized treatment [13]. Recovery must, for many young people, be understood as an ongoing process (progress) and a way of living rather than a return to the state before the suffering became too difficult to cope with [17,81,96,102,103].

## 7. Conclusions

The present study showed the importance of therapists actively using the case formulation in the treatment of depression. The study was carried out as an additional in-depth analysis of the qualitative data set from FEST-IT. The aim was, through qualitative methods, to achieve more particular findings or aspect than was undertaken as part of the primary quantitative analyses. Two representative patients with and without transference interpretations indicate how a more individualized approach can guide the therapy beyond diagnosis and make it more effective for the specific patient. Detailed focus on parts of a case formulation and the use of dynamic features in treatment may add more knowledge about what works for whom in short term psychotherapy. The focus on central dynamics as part of a case formulation may address the maintenance or precipitation of further depression. The therapists’ addressing of conflicted anger as a central dynamic of depression in two adolescents was successful. A firmly established therapy period provided the necessary stability in a confusing and difficult period in the adolescents’ life. As time passed within and between therapy sessions, the adolescents made experiences from which they, with crucial help from the therapist, were allowed to mature and grow.

The study of client experience during treatment may in different manners illuminate and expand service-user preferences. Preferred ways of working in therapy involve probabilistic inferences, and ongoing information from the patient’s experiences and progress in treatment may be necessary for essential adjustments. The therapist’s construction of a case formulation in collaboration with an evaluator as the most fitting mechanism(s) or CDD(s) may be critical. What client say about what he or she needs or wants, thinks, or feels may be decisive. Going forward, there is a need to focus on practice-based evidence in therapy, including large-scale routine outcome monitoring [70]. Such research can help to identify helpful and unhelpful aspects of therapy and puts the needs of young people and families at the heart of evidence-based practice [12]. Sensitivity and flexibility from the therapist may open up for a strong user involvement and a stronger working alliance.

We also need more attention towards potential adverse effects of treatment and the circumstances under which particular treatments and combinations of treatment might work best. The use of Core Critical Relational Themes (CCRT) in therapy describes the patient’s specific patterns of expectations on others and bring attention to the individual’s relationship patterns. Such information may scrutiny the impact from commonalities in treatment, but also how and why psychotherapy works or fails to work. Examining how the client and the therapist work together following a case formulation based on clinical information and psychodynamic theory may add to the growing knowledge base on individualized treatment. Picking out and putting together building pieces in a developmental story may strengthen and challenge dynamic features in personal development.

## Figures and Tables

**Table 1:** Pre-treatment characteristics of adolescents in an RCT offering psychoanalytical psychotherapy [35].

	Transference Work Group (n = 39)		Non-Transference Work Group (n = 30)	
	*N*	%	*N*	%
Gender				
Female	33	84.6	24	80.0
Male	6	15.4	6	20.0
Diagnostics (M.I.N.I *)				
Recurrent depression	15	38.5	9	30.0
Suicide risk (moderate to high)	6	15.4	4	13.3
Prevalence of one or more comorbid diagnoses	18	46.2	16	53.3
Age	Mean	(SD)	Mean	(SD)
	17.30	(0.7)	17.31	(0.7)
Personality diagnostics				
Average number of PD criteria (SIDP-IV **)	13.5	(9.0)	12.4	(7.8)

Notes * M.I.N.I; Mini International Neuropsychiatric Interview; ** SIDP-IV; Structured Interview for DSM-IV Personality.

## Data Availability

The data presented in this study are openly available in https://www.med.uio.no/klinmed/english/research/projects/fest-it/index.html (accessed on 14 June 2021).
